# Characterization and Localization of *Calb*2 in Both the Testis and Ovary of the Japanese Flounder (*Paralichthys olivaceus*)

**DOI:** 10.3390/ani10091503

**Published:** 2020-08-26

**Authors:** Yuting Xiang, Yahui Wu, Haoran Zhang, Jikui Wu, Junling Zhang

**Affiliations:** 1 Key Laboratory of Freshwater Aquatic Genetic Resources, Ministry of Agriculture; Key Laboratory of Exploration and Utilization of Aquatic Genetic Resources, Ministry of Education; Shanghai Collaborative Innovation Center for Aquatic Animal Genetics and Breeding; Shanghai Ocean University, Shanghai 201306, China; 15973236833@163.com (Y.X.); w17349711053@163.com (Y.W.); m17802590279@163.com (H.Z.); 2 Laboratory of Quality and Safety Risk Assessment for Aquatic Product on Storage and Preservation, Ministry of Agriculture; National R&D Branch Center for Freshwater Aquatic Products Processing Technology (Shanghai); Shanghai Ocean University, Shanghai 201306, China; 3 Laboratory for Marine Fisheries Science and Food Production Processes, Qingdao National Laboratory for Marine Science and Technology, Qingdao, Shandong 266071, China

**Keywords:** *calb*2, testis, ovary, *Paralichthys olivaceus*

## Abstract

**Simple Summary:**

Calretinin (CALB2), which is a Ca^2+^-binding protein, plays a known pivotal role in the neural system in vertebrates. The role of CALB2 in mammalian gonads has been gradually recognized; however, little information on the function of CALB2 in fish gonads has been reported. Therefore, we firstly identified the *calb*2 gene in *Paralichthys olivaceus* (*P. olivaceus*), and then investigated its tissue distribution and localization in the gonads by real-time PCR, western blotting, and immunohistochemistry. The *P. olivaceus calb*2 mRNA was relatively highly expressed in both the testis and ovary. The CALB2 protein is located in Leydig cells of the testis and ovarian germ epithelial cells in *P. olivaceus*. This study provides a basis for further explorations on the function and regulation mechanism of *calb*2 in fish gonads.

**Abstract:**

Although its function in mammalian gonads has been gradually recognized, the expression and function of calretinin (CALB2)—a Ca^2+^-binding protein—in the testis and ovary of fish are still unclear. Here, we identified the cDNA sequences of *calb*2 in *Paralichthys olivaceus* (*P. olivaceus*); analyzed its gene structure and phylogenetic and syntenic relationship by bioinformatics; and investigated its tissue distribution and localization in the gonads by real-time PCR, western blotting, and immunohistochemistry. The *P. olivaceus* *calb*2 gene has 11 exons and 10 introns, and the full-length cDNA is 1457 bp, including an open reading frame (ORF) of 816 bp encoding 271 amino acids. The CALB2 of *P. olivaceus* has a higher homology with *Lates calcarifer* (99%) compared with other species. The conserved synteny of *calb*2 neighboring gene loci was also detected in fish. Real-time PCR showed that the expression of *calb*2 mRNA is abundant not only in the brain, but also in the gonads, and exhibits a higher expression in the testis than in the ovary. Western blotting indicated that the CALB2 protein has a higher expression in the testis compared with the ovary. Immunohistochemistry demonstrated that the CALB2 protein appears in Leydig cells and the ovarian germ epithelium. These results reveal that *calb*2 plays an important role in the gonads of *P. olivaceus*.

## 1. Introduction

Calretinin (CALB2) is a Ca^2+^-binding protein, with a typical EF-hand calcium binding domain that can be reversibly combined with Ca^2+^ as a calcium receptor. CALB2, together with parvalbumin and calbindin-28kD, belongs to the superfamily of Ca^2+^-binding proteins, and was first discovered by Rogers in the retina of a chicken [1,2,3]. The main role of CALB2 is as a sensor and buffer of intracellular Ca^2+^ to prevent Ca^2+^ overload [4]. Ca^2+^ is an important secondary messenger, activating downstream protease via the PKC or CAM pathway, and CALB2 is thus involved in skeletal muscle/myocardial contraction, nerve reaction impulse conduction, steroid hormone synthesis and application, and endocrine and immune systems. It also plays an important role in cell differentiation, development, proliferation, necrosis, and apoptosis [5,6,7]. For example, after 5-fluorouracil (5-FU) treatment of the HCT116 CRC cell line, CALB2 was involved in apoptosis induction through the inherent mitochondrial pathway, suggesting that CALB2 may be an important mediator of 5-Fu-induced cell death [7]. Recently, newly identified lncRNA CALB2 was found to regulate RUNX2 expression as a Mir-30b-3P sponge, thus promoting the differentiation of hDPSCs odontoblast cells, and the LncRNA CALB2/Mir-30b-3P/RUNX2 axis may be a new therapeutic target for promoting odontogenesis [8].

Calretinin is mainly expressed in the nerve center and surrounding nerve tissues [9,10]. Although usually considered to be freely diffusible and uniformly distributed in the cytoplasm, at a certain development stage, it has been shown to be highly concentrated under the cell membrane [11]. In mammals, calretinin is also expressed in the ovaries and testes, which can synthesize steroid hormones [1,12]. In normal ovarian tissue, calretinin is detected in the endometrial cells of the follicles, including the secondary follicle, vesicular follicle, corpus luteum, corpus luteum of pregnancy, and visible ovarian surface epithelial cells. However, it is not present in oocytes or granulosa cells. Calretinin may regulate the synthesis of hormones by directly affecting the secretion of stromal cells or indirectly affecting the sensory neurons and paravertebral nerve of the ovary [1]. Moreover, calretinin is positively expressed in Leydig cells, cytoplasm, supported cells, and testicular membrane cells, and is negatively expressed in sperm cells and the epididymal epithelium [12]. Calretinin is also highly expressed in testicular mass cell tumors and associated with the promotion of interstitial cell growth and the inhibition of cell apoptosis [13,14]. In zebrafish (*Danio rerio*), calretinin is widely expressed in the central nervous system [15,16] and chemosensory cells [17]. And calretinin is also present in the peripheral nervous system of zebrafish [18], and plays an important role in the development of the eye, brain, spinal cord, and other tissues during early embryonic development [19]. It was also found that the combined knockout of *calb*2*a* and *calb*2*b* was associated with an impaired touchdown and swimming performance of zebrafish [20]. The distribution of calretinin at a protein level in the brain was reported in an “advanced” teleost (*Chelon labrosus*), indicating that the neural system using calretinin to maintain the intracellular calcium concentration has been rather conservative in vertebrate evolution [21]. Moreover, calretinin was expressed in the pituitary gland in the hardhead catfish (*Arius felis*), revealing that it can participate in hormonal regulation [22]. However, little is known about the expression pattern and potential role of *calb*2 in fish gonads.

The Japanese flounder (*Paralichthys olivaceus* (*P. olivaceus*)) is an important commercially farmed marine flatfish; the female of the species grows faster than the male. Its sex determination mainly depends on genetic and environmental factors. Therefore, investigation of the molecular regulatory mechanism of sex differentiation and gonadal development in this species is of great significance in aquaculture. In this study, we identified the cDNA sequences of *calb*2 in *P. olivaceus*; analyzed its gene structure and phylogenetic and syntenic relationship by bioinformatics; and examined its tissue distribution by real-time PCR and western blotting, and localization in the gonads by immunohistochemistry. The findings will contribute to the understanding of the role of *calb*2 in the gonads of *P. olivaceus*.

## 2. Materials and Methods

### 2.1. Experimental Fish

The *P. olivaceus* adults were acquired from the Beidaihe Central Experimental Station, Hebei, China. The weight of the one-year female fish was 805 ± 12 g, and that of the male fish was 555 ± 8 g. The tissues, including those from the brain, ovary, testis, gill, liver, heart, kidney, muscle, stomach, and intestine, were dissected randomly from three female and three male fish (*n* = 3). All samples were swiftly frozen in liquid nitrogen and stored at −80 °C until RNA and protein extraction. In addition, the ovary and testis were cut and fixed in Bouin and 4% paraformaldehyde for 24 h, and then transferred into 70% ethanol and stored at 4 °C for Hematoxylin and Eosin (HE) staining and immunohistochemistry. All animal protocols were approved by the Shanghai Ocean University Review Committee for the Use of Animal Subjects (Shou-DW-2018-010).

### 2.2. RNA Extraction, Reverse Transcription, and Molecular Cloning

RNA from all samples was extracted by the Trizol Reagent method (Invitrogen, Carlsbad, CA, USA). The OD260/280 value and concentration were determined by NanoDrop 2000 (Thermo, Woltham, MA, USA). The RNA integrity of each sample was detected by 1% agarose gel electrophoresis and then stored at −80 °C.

RNA was reverse-transcribed after treatment with DNase I as follows: Total RNA of 1000 ng and OligodT Primer (50 μM) of 1 μL, supplemented with ddH_2_O until reaching 10 μL, at 70 °C for 10 min, and then placed on ice immediately for 5 min. The following reagents were added: 5 × M-MLV buffer, 5 μL; Rnase Inhibitor (25 U), 1 μL; M-MLVRtase (200 U, Promega, Madison, Wisconsin, USA), 1 μL; dNTP mixture (10 μM), 1 μL; and ddH_2_O until reaching 10 μL. The reverse transcription reaction was run at 42 °C for 60 min and 75 °C for 15 min. The obtained cDNA was stored at −20 °C.

By searching the NCBI database, we obtained the predicted cDNA sequences encoding the *P. olivaceus calb*2 gene (XM_020106004.1). The cDNA sequence of *calb*2 was verified by PCR cloning and sequencing. The primers of *calb*2 were designed on Primer Premier 5.0 software for cloning. The primers were synthesized by the Invitrogen (Shanghai) company and the sequences are described in Table 1.

### 2.3. Bioinformatics Analysis

The cloned cDNA fragments of the *P. olivaceus calb2* gene were spliced with DNAMAN software. Then, the physicochemical properties of the predicted proteins were analyzed online using the ExPASy-ProtParam tool. The genome and transcriptome of *P. olivaceus* have been published by Shao [23]. The sequences of *P. olivaceus calb*2 and its counterparts from other vertebrates were introduced into the Gene Structure Display System (GSDS, http://gsds.cbi.pku.edu.cn/), in order to map the gene structure. Multiple sequence alignment was performed for *P. olivaceus* CALB2 and its homologous proteins using BioEdit software. The phylogenetic tree was constructed by MEGA 5.2. The GenBank accession number of the sequences used are *Danio rerio* (NP_957012.1), *Oreochromis niloticus* (XP_003439492.1), *Oryzias latipes* (XP_004069798.1), *Oncorhynchus mykiss* (XP_021453615.1), *Xenopus tropicalis* (XP_012817093.1), *Pogona vitticeps* (XP_020653551.1), *Rattus norvegicus* (NP_446440.1), *Gallus gallus* (NP_990647.1), *Macaca mulatta* (EHH31827.1), *Homo sapiens* (CAA39991.1), *Boleophthalmus pectinirostris* (XP_020790837.1), *Lates calcarifer* (XP_018559013.1), *Esox lucius* (XP_010878050.1), and *Mus musculus* (NP_031612.1). For syntenic analysis, the orientation and chromosomal position of the *calb*2 gene and its adjacent genes were determined through the Ensembl Genome Browser (http://www.ensembl.org/index.html).

### 2.4. Real-Time PCR

The gene-specific quantitative primers (Table 1) were designed using Primer5.0 software. Real-time PCR was run on the CFX96 Touch^TM^ Real Time PCR Detection System (Bio-Rad, Hercules, CA, USA). To estimate the amplification efficiencies, the standard curves were generated for the target gene (*calb*2) and internal standard gene (18 *s*). According to the instruction manual of SYBR^®^ Premix Ex Taq™ II (TaKaRa, Shiga, Japan), the procedure of two-step PCR amplification was performed. The reaction system (20 μL) was 1 μL cDNA (100–0.8 ng, five times gradient dilution), 0.4 μL of each of the forward and reverse primers, 10 μL SYBR^®^ Premix Ex Taq™ II, and 8.2 μL ddH_2_O. The amplification protocol was 95 °C for 10 min, followed by 39 cycles of 95 °C for 10 s and 60 °C for 30 s, including annealing and extension accompanied by the acquisition of fluorescence and the reading of melt curve data. All calibration curves exhibited correlation coefficients higher than 0.99, and the corresponding efficiencies (E) of PCR ranged from 0.95 to 0.99. The real-time PCR reaction system and procedures of all samples were the same as presented above. The internal standard (18 s) and target gene (*calb*2) were employed in a side-by-side manner and each experiment was repeated in triplicate.

The relative expression for *calb*2 mRNA was calculated by the comparative threshold method (2^−ΔΔCt^) [24], and the liver was taken as a control tissue. The results were indicated by means ± standard deviation (SD). Statistical analysis was performed with one-way of variance (ANOVA) using SPSS 17 software, and group effects were further investigated by the Dunnett-T3 test. Differences were statistically significant at *p* < 0.05.

### 2.5. Western Blotting

The testes and ovaries, rinsed with PBS, were immersed in precooled cell lysis solution (containing phenylmethylsulfonyl fluoride, PMSF, 1 mM, biotechwell, Shanghai, China) in a 1.5 mL centrifuge tube. Tissue cells were disrupted with a homogenizer (TIANGEN, Beijing, China), which was followed by the addition of ice for a few minutes and repeated several times, so that the cells were disrupted as much as possible. The suspension was centrifuged (12,000 rpm, 4 °C, 5 min), and the supernatant was preserved at −20 °C for western blot analysis.

The supernatant was mixed with SDS-PAGE protein loading buffer 5X (1:4; Sangon Biotech, Shanghai, China) and then placed in a boiling water bath for 5 min. Proteins (80 ng, 15 μL) extracted from testis and ovarian tissues and EasySee Western Marker (20–90 kDa, 5 μL, transgenbiotech, Beijing, China) were loaded onto SDS-PAGE for electrophoresis (sodium dodecyl sulfate polyacrylamide gelelectropheresis). The initial voltage was 80 V for 20 min, and was then increased to 120 V. The voltage was terminated when the bromphenol blue started to run out. After separation, proteins were blotted onto a nitrocellulose membrane (15 V, 30 min) and blocked in 5% skimmed milk preparation with TBST for 2 h. They were incubated overnight at 4 °C with the CALB2 Polyclonal Antibody (Abclonal) and Anti-ACTB rabbit polyclonal antibody (BBI Life Sciences Corporation, Shanghai, China) at a dilution of 1:1000. The secondary antibody—HRP-conjugated Goat Anti-Rabbit IgG (BBI Life Sciences Corporation)—was diluted to 1:2000 with TBST and incubated at room temperature for 1 h. For detection, the ECL luminescence reagent (Sangon Biotech, Shanghai, China) was used. Chemiluminescent and digital images were recorded on ImageQuant™ LAS 4000 (GE Healthcare life sciences, Chicago, IL, USA).

### 2.6. HE Staining and Immunohistochemistry

Tissue specimens of the testis and ovary fixed in Bouin were cut into 0.2 cm pieces, and were dehydrated using graded ethanol, vitrified by dimethylbenzene, suffused with wax at 65 °C, and embedded using paraffin. Archival paraffin blocks of tissue were serially sectioned into samples with a thickness of 5 μM. Continuous baking was maintained for 12–48 h at 37 °C. Further de-waxing was performed by dimethylbenzene and rehydrated by ethanol. The nucleus and cytoplasm were stained with hematoxylin and eosin, respectively. Finally, neutral resins were added to the tissue surface after dehydration and vitrification. The sections from testes and ovaries were observed microscopically.

Testis and ovary tissues fixed in 4% paraformaldehyde were treated with the immunohistochemical method: Tissues were incubated in 3% hydrogen peroxide (H_2_O_2_) for 10 min at room temperature to cease endogenous peroxidase activity and washed with PBS for 5 min × 3 times; antigen repair was performed at room temperature for 10 min and flushed with PBS for 10 min × 2 times; tissues were blocked with normal goat serum (Bosterbio, Pleasanton, CA, USA) for 30 min; specimens were incubated overnight at 4 °C with the CALB2 Polyclonal Antibody (Abclonal, Woburn, MA, USA) (negative control was replaced by PBS) at 1:100 dilution; after washing, tissues with coverslips were incubated with Biotin-labeled Goat anti-rabbit IgG (1:1000, Bosterbio) for 30 min; the immune response was enhanced by SABC for 30 min at room temperature, and then flushed with PBS for 10 min × 3 times; visualization was performed using DAB-kit-Pale-brown (Bosterbio) under the microscope to observe coloring to facilitate the termination reaction; and tissues were flushed with water for 5–10 min. The steps followed for dehydration and mounting were the same as for HE. Photographs were taken under a light microscope (Nikon, Minato, Tokyo).

## 3. Results

### 3.1. Molecular Characterization and Phylogenetic and Syntenic Analyses of Calb2

**Figure 1:**
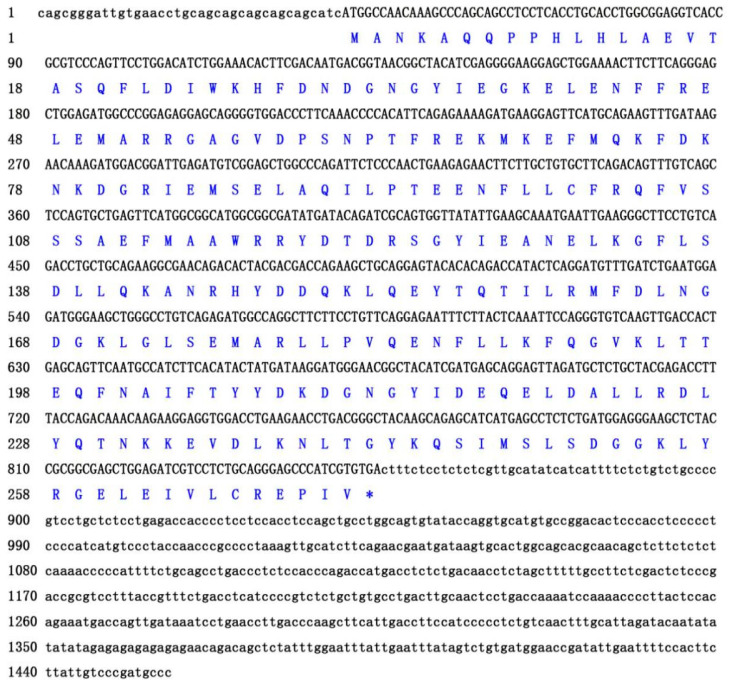
The cDNA and amino acid sequences of *calb*2 in *Paralichthys olivaceus.* * indicates a stop codon.

**Figure 2:**
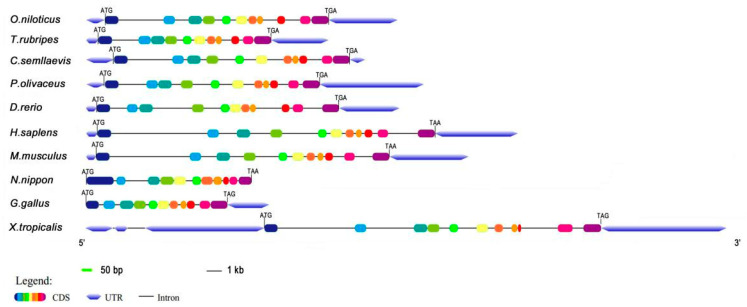
Gene structure of the *P. olivaceus calb*2 and its counterparts from other vertebrates. The color frame and black line represent exons and introns, respectively. ATG and TAG (TGA, TAA) indicate a start and stop codon, respectively.

The obtained full-length cDNA of *P. olivaceus calb*2 is 1457 bp, including an open reading frame (ORF) of 816 bp encoding 271 amino acids (Figure 1). The deduced protein has an estimated molecular mass of 31.52 kDa and a predicted isoelectric point (PI) of 5.08. As shown in Figure 2, the *calb*2 gene from *P. olivaceus* and its counterparts from other vertebrates have 11 exons and 10 introns. The sequence length and structure distribution of the *P. olivaceus calb*2 gene are similar to evolutionarily close species.

**Figure 3:**
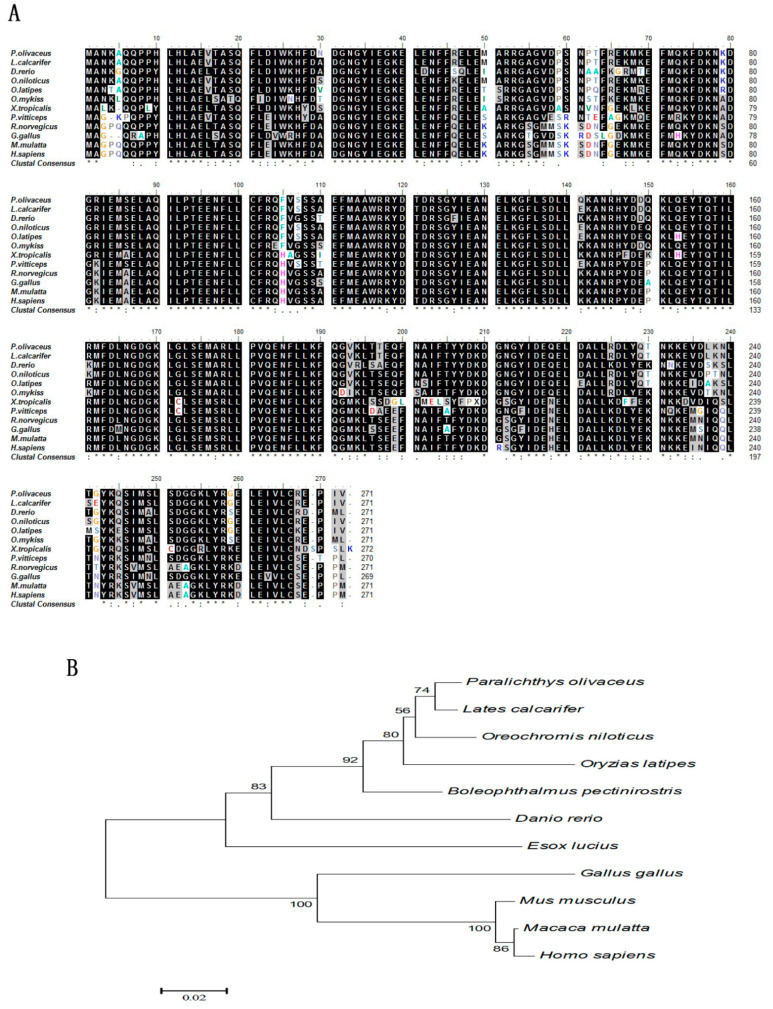
Molecular phylogenetic analysis of CALB2. (**A**) Amino acid sequence alignment of the *P. olivaceus* CALB2 with that of other species (light black blocks show identical amino acid residues for the sequences shown); (**B**) phylogenetic tree of the *P. olivaceus* CALB2 relative to the homologues from other vertebrates (bootstrap values are shown at the branch points).

Alignment analysis revealed that the CALB2 of *P. olivaceus* has the highest homology with that of *L. calcarifer* (99%) compared with other species (Figure 3A), followed by *O. niloticus* (97%), *O. latipes* (93%), *O. mykiss* (89%), and *D. rerio* (87%). The homology is lower compared with amphibians, reptiles, and mammals; for example, the homology with *X. tropicalis* is 78%, and that of *P. vitticeps*, *R. norvegicus*, *M. mulatta*, and *H. sapiens* is 79%. However, homologous domains were shown to be highly conservative in vertebrates. The phylogenetic tree constructed by Neighbor-Joining showed that the *P. olivaceus* CALB2 protein is located in the same group as all fish (Figure 3B).

**Figure 4:**
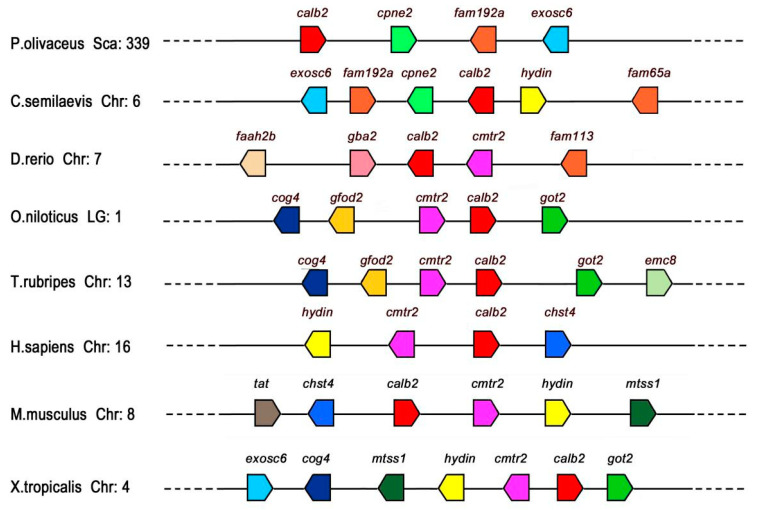
Synteny maps for the *P. olivaceus calb*2 on the scaffold and its counterparts from other vertebrate chromosomes constructed using the Ensembl Genome Browser (http://www.ensembl.org). The bar lengths are not proportional to the distances between genes. Gene symbols were determined by Browser. The direction of the arrows indicates the gene orientation. Chr, chromosome; LG, linkage group; Sca, scaffold.

To elucidate the conservatism of *calb*2 and adjacent genes between *P. olivaceus* and other vertebrates (Figure 4), this study compared the *calb*2 gene loci of *P. olivaceus* and other vertebrates on chromosomes (Chr) or linkage groups (LGs). The *P. olivaceus calb*2 gene located in scaffold339 and *cpne*2, *fam*192*a*, and *exosc*6 genes coexisted with *calb*2 in most teleosts, and the three genes were located downstream of *calb*2 in *P. olivaceus*, *O. latipes*, and *O. niloticus*, while they were upstream of *calb*2 in *Cynoglossus abbreviatus*. The *cog*4 and *cmtr*2 genes existed upstream of *calb*2 in *O. niloticus*, *T. rubripes*, *H. sapiens*, *X. tropicalis*, and *M. musculus*. In general, conserved synteny of *calb*2 neighboring gene loci was detected in fish rather than amphibians and mammals.

### 3.2. Tissue Distribution of calb2 mRNA

**Figure 5:**
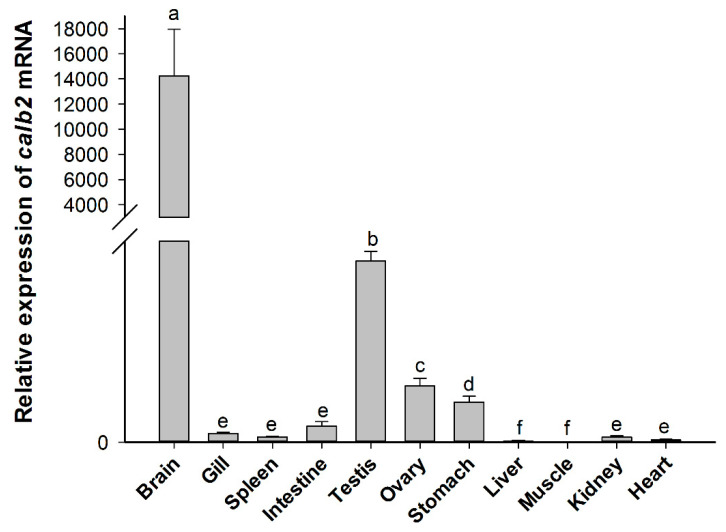
Relative expression levels of *calb*2 mRNA in different tissues from *P. olivaceus*. Each gene was calculated based on the level of expression in the liver. The different alphabetic symbols represent statistically significant differences (*p* < 0.05).

The spatial distribution of *calb*2 mRNA in adult *P. olivaceus* is shown in Figure 5. The real-time PCR results showed that *calb*2 is widely expressed in all adult tissues investigated. The highest level of *calb*2 was detected in the brain, and its expression was extremely high compared with other tissues. A higher level was present in the testis and ovary, while a lower level was observed in the stomach, and only a very low expression was found in the intestine, spleen, gill, heart, liver, and muscle. Moreover, there was a significant difference in the expression between the testis and ovary (*p* < 0.01). The quantitative results showed that the expression level of *calb*2 in the testis is about three times that of the ovary.

### 3.3. Expression and Localization of the CALB2 Protein in the P. olivaceus Gonads

**Figure 6:**
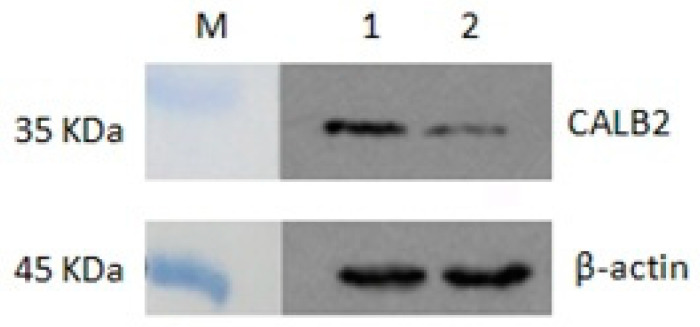
Expression analysis of the CALB2 protein in the testis and ovary of *P. olivaceus* by western blot analysis. M, protein marker; 1, testis; 2, ovary.

Western blot analysis with proteins isolated from adult *P. olivaceus* gonads revealed a specific strong signal in the position near 32 kDa (Figure 6). This result was consistent with the predicted molecular weight of CALB2, and the expression of CALB2 in the testis was relatively higher than that in the ovary.

**Figure 7:**
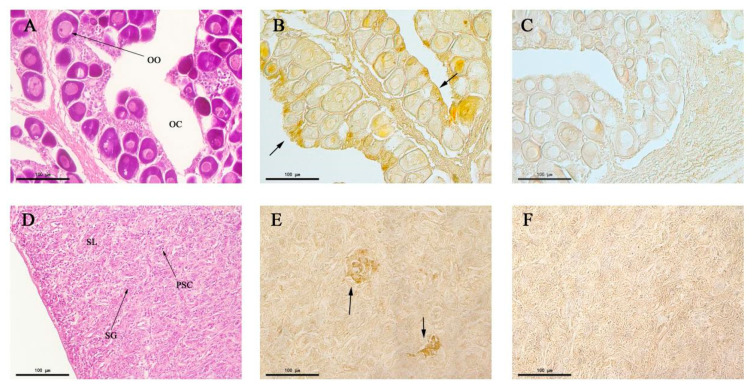
Localization of the CALB2 protein in the testis and ovary of *P. olivaceus* detected by immunohistochemistry. (**A**) Ovarian tissue section with Hematoxylin and Eosin (HE) staining; (**B**) CALB2 immunoreactivity in the ovary; (**C**) negative control of B; (**D**) testis tissue section with HE staining; (**E**) the CALB2 immunoreactivity in the testis; (**F**) negative control of E. OO, oocytes; OC, ovarian cavity; SG, spermatogonium; SL, seminal lobule; PSC, primary spermatocyte.

The structures of the testis and ovary of *P. olivaceus* were observed through paraffin section and HE staining (Figure 7A,D). The spawning plates of the ovary were filled with stage II primary oocytes, with a round or irregular ellipse shape, and the cytoplasm was homogeneous. Transparent oil balls appeared in the near nuclear membrane of some oocytes (Figure 7A). The inside of the testis was divided into many seminal lobules by connective tissue; lobules were tightly packed, and the spermatogonium was round or elliptical, with a weakly stained cytoplasm (Figure 7D). Immunohistochemistry showed the expression of CALB2 in the ovarian germ epithelium (Figure 7B). However, no obvious immune signals were detected in oocytes. There were positive signals in the mesenchymal cells from the seminiferous lobules in the testis (Figure 7E).

## 4. Discussion

CALB2 belongs to the superfamily of CaBPs and is encoded by the *calb*2 gene in humans [25]. It is an important active substance in nerve cells as a buffer of intracellular Ca^2+^. Based on the genome and transcriptome analysis of *P. olivaceus* by Shao [23], we first identified the cDNA sequences of the *calb*2 gene in *P. olivaceus* and investigated the homology and synteny of *calb*2 in different species by gene structure analysis, multiple sequence alignment, phylogenetic trees, and gene collinearity expression. The Gene Structure Display System demonstrated that the structure of the *calb*2 gene in *P. olivaceus* is similar to that in other species. The CALB2 complete sequences encoded about 270–289 amino acids and contained the same number of exons, though there were differences in the UTR region and intron, and the CALB2 had six typical EF-hand calcium binding domains, but one did not show Ca^2+^ affinity [26,27]. Moreover, alignment analysis showed that the amino acid sequences of CALB2 have the highest homology with *L. calcarifer* (99%), followed by *O. niloticus* (97%), *O. latipes* (93%), and *O. mykiss* (89%). Phylogenetic analysis revealed that *P. olivaceus* CALB2 is located in the same group as *L. calcarifer*, and the evolutionary relation with *P. olivaceus* CALB2 is close to other fish, but distant from humans and mice. Syntenic analysis also proved the conserved synteny of *calb*2 and its neighboring genes in the teleosts. The above analysis shows that the CALB2 protein homeodomain and the typical CALB2 domain are relatively conserved in the phylogenetic evolution of species.

In the present study, an exceedingly high expression of *calb*2 mRNA was observed in the brain of adult *P. olivaceus*, and similar results have also been reported in zebrafish [19]. A very high expression of *calb*2 mRNA was noted in the tegmentum, midbrain-hindbrain boundary, cranial ganglion, neural crest, etc. in embryonic development, suggesting that *calb*2 plays a pivotal role in neural system development. In teleosts, calretinin was widely distributed in the central nervous system [15,16] and chemosensory cells [17], and also existed in the peripheral nervous system [18], which is helpful for completing its distribution map in the teleost nervous system. In freshwater catfish (*Clarias batrachus*), a sexually dimorphic distribution of calretinin has been shown in the preoptic area (POA), which plays an important role in the regulation of pituitary hormones in vertebrates. This indicated that calretinin may have a sex-specific effect and may be involved in the regulation of fish hormones [28]. However, little information on the expression of *calb*2 has been reported in fish gonads. In this study of *P. olivaceus*, *calb*2 mRNA was also expressed at high levels in the adult gonads, and its expression was higher in the testis than in the ovary. This difference of expression may be related to the *calb*2 function in the testis and ovary.

Based on these results, the differential expression of the CALB2 protein in the testis and ovary of adult *P. olivaceus* was further verified. Western blot analysis showed that the molecular weight of the CALB2 protein is 31.5 kD and its expression in the testis is higher than that in the ovary. This is consistent with the expression of *calb*2 mRNA in the testis and ovary of *P. olivaceus*. Immunohistochemistry results displayed that positive signals for CALB2 appeared in Leydig cells of the testis and also ovarian germ epithelial cells in *P. olivaceus*. Similar results have been reported in rats [29], CALB2 was mainly located in the cytoplasm of Leydig cells in testicular tissue, whereas its immunoreactivity was weak in Sertoli cells and completely negative in spermatogenic cells. In addition, CALB2 presented a differential expression at different sexual developmental stages. Compared to the pre-sexual maturity and older stages, the expression of CALB2 in the testes of rats was significantly increased during sexual maturity and positively correlated with the serum testosterone level, indicating that CALB2 was involved in regulating testosterone production [29]. In humans, calretinin was detected in adult and fetal testes and was visible in fetal Leydig cells throughout the 14th to 27th week. Furthermore, calretinin was also observed in Sertoli cells and in the germ cells of immature seminiferous tubules of fetal testes, which could be linked to the ability of Sertoli cells to produce locally acting hormones. However, the expression of calretinin was parallel to the decrease in the Leydig cell number, suggesting that calretinin is indeed correlated with its steroidogenic activity [30]. Moreover, the highest expression of calretinin was also found in Leydig cells of the adult testis, and various studies have provided strong evidence that calretinin is involved in androgen synthesis in Leydig cells [14,31,32].

In the present study, CALB2 immunoreactivity was observed in ovarian germ epithelial cells of *P. olivaceus*. Similarly, a strong positive immunoreactivity of calretinin was found in the ovarian surface epithelium of normal women [33]. It has been reported that calretinin was specifically localized in human ovarian germinal epithelial cells and also present in a few cells of the theca externa and some interstitial cells [1]. In rat ovaries, calretinin was expressed at both mRNA and protein levels from 19 days after birth to the adult stage, but not after 10 days. More significantly, calretinin was limited to the interstitial tissue, and its protein and mRNA did not appear in other ovarian regions [34]. Previous studies have shown that Ca^2+^ plays an important role in hormone production, and the expression of CALB2 suggests that it may regulate hormone production by regulating the concentration of Ca^2+^. However, the biological mechanism of CALB2 in the ovary is not known.

More importantly, testicular Leydig cells are testosterone-producing cells that have many neuroendocrine properties and are part of a diffuse neuroendocrine system [35]. The pituitary luteinizing hormone (LH) controls steroidogenesis and the process of testosterone secretion from Leydig cells, which is in part influenced by intracellular calcium levels [6]. In addition, some studies have demonstrated that alterations in intracellular calcium concentrations affect Leydig cell steroidogenesis [36,37]. It has been found that the over-expression of calretinin in MLTC-1 cells up-regulated the level of testosterone, while the level of progesterone was significantly decreased in R2C cells due to the inhibition of calretinin by siRNA-*calb*2 [33]. Briefly, these results suggest that *calb*2 plays an important role in gonads via regulating the steroidogenesis in vertebrates.

## 5. Conclusions

In summary, we first identified the cDNA sequences of the *calb*2 gene in *P. olivaceus*. The expression analysis of the *P. olivaceus calb*2 mRNA showed that it is relatively highly expressed in the brain and gonads, and has a significantly different expression in the testis compared to the ovary. Immunohistochemistry results revealed that the CALB2 protein is located in Leydig cells of the testis and ovarian germ epithelial cells in *P. olivaceus*. This study provides a theoretical basis for further studies on the regulation mechanism of *calb*2 in the *P. olivaceus* gonads.

## Figures and Tables

**Table 1:** The primer sequences for cloning and real-time quantitative PCR of *calb*2.

Primer	Primer Sequence (5′→3′)
*calb2*-F1 Primer	AGCCCAGCAGCCTCCTCA
*calb2*-R1 Primer	CTTGTAGCCCGTCAGGTTCT
*calb2*-F2 Primer	GGACCTGAAGAACCTGACGG
*calb2*-R2 Primer	GGGCATCGGGACAATAAGAA
*calb2*-F3 Primer	TAAAAGCGGCGGGCGGTGCG
*calb2*-R3 Primer	GAATGTGGGGTTTGAAGGGT
*calb2*-F4 Primer	TATCAGCGGGATTGTGAACC
*calb2*-R4 Primer	GAATGTGGGGTTTGAAGGGT
q*calb*2-F	CAGAAGGCGAACAGACACTACG
q*calb*2-R	GGTCAACTTGACACCCTGGAAT
q*18s*-F	CTTAGTTGGTGGAGCGATTTG
q*18s*-R	CTCGGCGAAGGGTAGACA
